# Supplementary figures and images for: Seed priming with essential oils for sustainable wheat agriculture in semi-arid region
Source: PeerJ. 2023 Mar 27;11:e15126. doi: 10.7717/peerj.15126 (PMC10062347; doi:10.7717/peerj.15126)

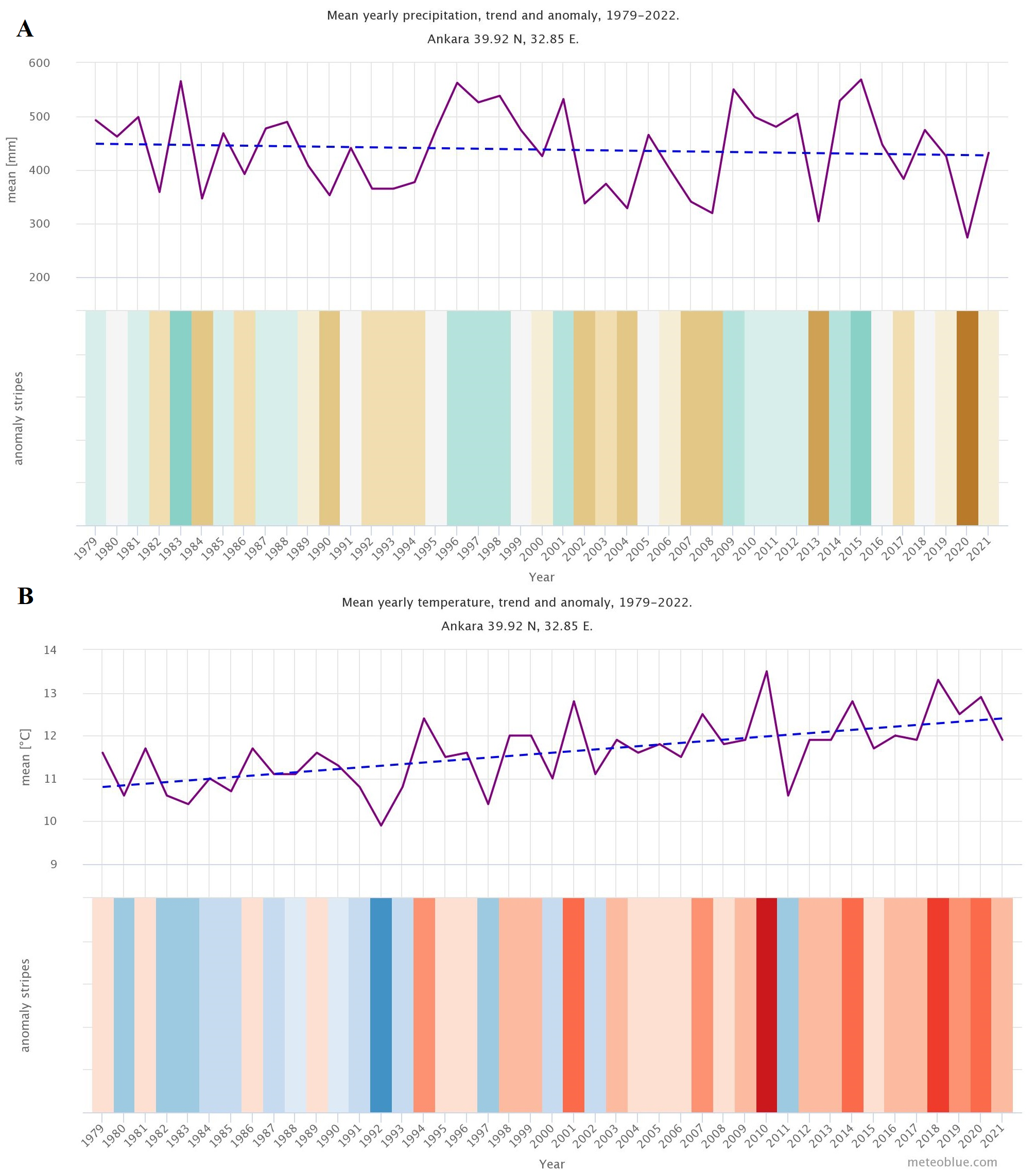

Supplement: Supplemental Information 1 [file peerj-11-15126-s001.png]

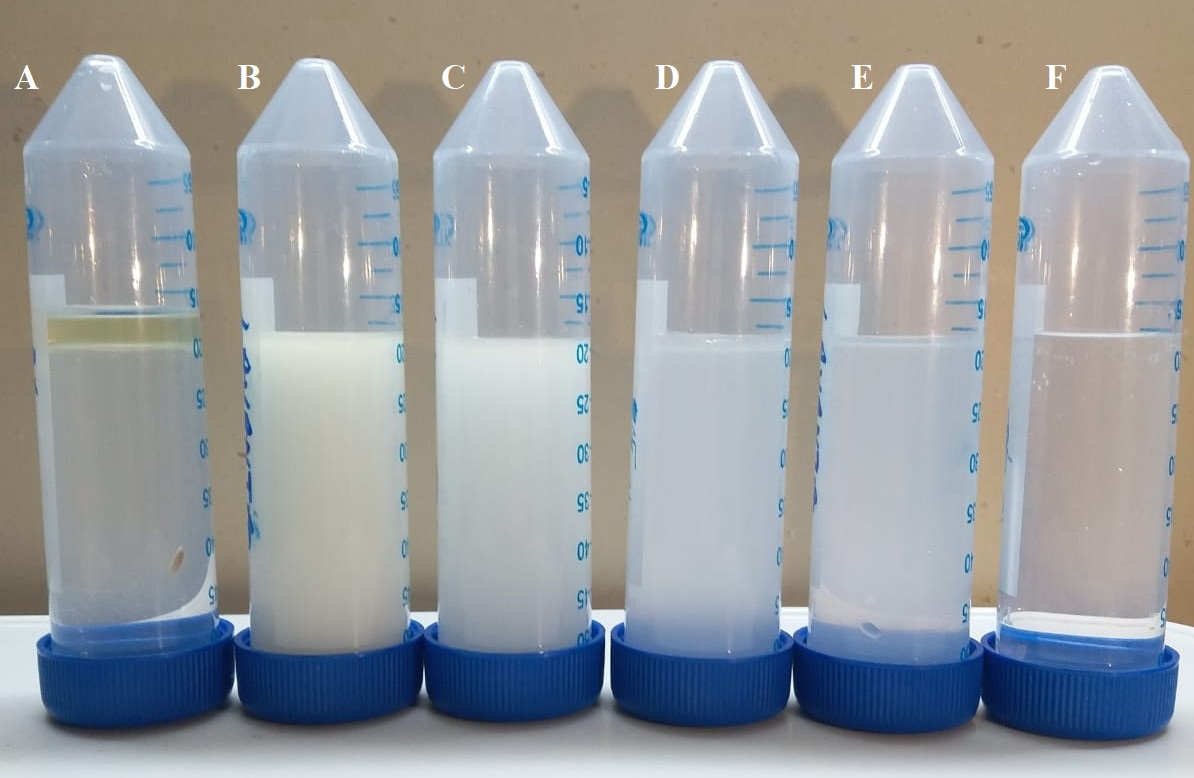

Supplement: Supplemental Information 2 — (A) Image of stock essential oil emulsion in water. (B) 0.25% solution prepared by dilution from stock. (C) 0.10% solution prepared by dilution from stock. (D) 0.05% solution prepared by dilution from stock. (E) 0.01% solution prepared by dilution from stock. (F) 0% control solution. [file peerj-11-15126-s002.png]
