# Supplementary material for: Seed priming with essential oils for sustainable wheat agriculture in semi-arid region
Source: PeerJ. 2023 Mar 27;11:e15126. doi: 10.7717/peerj.15126 (PMC10062347; doi:10.7717/peerj.15126)
Supplement: Supplemental Information 4 [file peerj-11-15126-s004.docx]

Tables created according to the statistical analysis results of Figures 2,3,4,5 and 6 are given below. In addition, the raw data entered for statistical analysis were shared in the "Raw data laboratory experiment" file.

| Germination | Rosemary | Sage | Lavander | Total |
| --- | --- | --- | --- | --- |
| D_0_ | 91,4000 | 91,0000 | 90,6000 | 91,0000^b^ |
| D_1_ | 89,4000 | 94,0000 | 89,4000 | 90,9333^b^ |
| D_2_ | 93,3000 | 94,0000 | 92,4000 | 93,2333^a^ |
| D_3_ | 81,6000 | 85,4000 | 78,4000 | 81,8000^c^ |
| D_4_ | 41,7000 | 40,9000 | 40,9000 | 41,1667^d^ |
| Total | 79,4800^b^ | 81,0600^a^ | 78,3400^c^ |  |

*The differences between the averages shown with different letters in the columns and rows are significant at the rate of 0.01.

*D_0_ (control), D_1_: 0.01%, D_2_ : 0.05%, D_3_ : 0.10%, D_4_ : 0.25%

| Coleoptile length | Rosemary | Sage | Lavander | Total |
| --- | --- | --- | --- | --- |
| D_0_ | 5,1500 | 5,6500 | 4,9000 | 5,2333^b^ |
| D_1_ | 6,4500 | 7,1500 | 5,6000 | 6,4000^a^ |
| D_2_ | 7,1000 | 6,6000 | 5,4000 | 6,3667^a^ |
| D_3_ | 2,3000 | 5,0000 | 4,6000 | 3,9667^c^ |
| D_4_ | 2,3000 | 2,1000 | 2,0500 | 2,1500^d^ |
| Total | 4,6600^b^ | 5,3000^a^ | 4,5100^b^ | 4,8233 |

*The differences between the averages shown with different letters in the columns and rows are significant at the rate of 0.05.

| Shoot length | Rosemary | Sage | Lavander | Total |
| --- | --- | --- | --- | --- |
| D_0_ | 17,0500 | 17,0500 | 16,9000 | 17,0000^b^ |
| D_1_ | 18,7000 | 20,4500 | 14,5500 | 17,9000^ab^ |
| D_2_ | 22,4000 | 19,2000 | 13,9500 | 18,5167^a^ |
| D_3_ | 19,5000 | 14,5500 | 13,6500 | 15,9000^c^ |
| D_4_ | 11,0000 | 9,4000 | 11,7000 | 10,7000^d^ |
| Total | 17,7300^a^ | 16,1300^b^ | 14,1500^c^ |  |

*The differences between the averages shown with different letters in the columns and rows are significant at the rate of 0.01.

| Root length | Rosemary | Sage | Lavander | Total |
| --- | --- | --- | --- | --- |
| D_0_ | 9,9500 | 9,8000 | 10,9500 | 10,2333^c^ |
| D_1_ | 12,5500 | 14,0000 | 11,3500 | 12,6333^b^ |
| D_2_ | 13,7000 | 15,2500 | 12,3500 | 13,7667^a^ |
| D_3_ | 14,3000 | 14,1000 | 9,5500 | 12,6500^b^ |
| D_4_ | 10,2000 | 11,1000 | 9,3500 | 10,2167^c^ |
| Total | 12,1400^a^ | 12,8500^a^ | 10,7100^c^ | 11,9000 |

*The differences between the averages shown with different letters in the columns and rows are significant at the rate of 0.01.

| Proline | Rosemary | Sage | Lavander | Total |
| --- | --- | --- | --- | --- |
| D_0_ | 2,4900 | 2,4200 | 2,1900 | 2,3667^a^ |
| D_1_ | 2,4600 | 2,5100 | 2,0100 | 2,3267^b^ |
| D_2_ | 2,0500 | 2,2400 | 2,2400 | 2,1767^e^ |
| D_3_ | 2,0900 | 2,3900 | 2,2000 | 2,2267^d^ |
| D_4_ | 2,5200 | 2,1000 | 2,0800 | 2,2333^c^ |
| Total | 2,3220^b^ | 2,3320^a^ | 2,1440^c^ | 2,2660 |

*The differences between the averages shown with different letters in the columns and rows are significant at the rate of 0.01.

| RWC | Rosemary | Sage | Lavander | Total |
| --- | --- | --- | --- | --- |
| D_0_ | 70,3860 | 69,3490 | 69,9220 | 69,8857^c^ |
| D_1_ | 71,0480 | 70,2320 | 70,0790 | 70,4530^b^ |
| D_2_ | 75,3210 | 71,9170 | 71,4830 | 72,9070^a^ |
| D_3_ | 71,3720 | 67,6780 | 69,3260 | 69,4587^d^ |
| D_4_ | 70,8650 | 63,9950 | 68,0140 | 67,6247^e^ |
| Total | 71,7984^a^ | 68,6342^c^ | 69,7648^b^ | 70,0658 |

*The differences between the averages shown with different letters in the columns and rows are significant at the rate of 0.01.

| Chlorophlly total | Rosemary | Sage | Lavander | Total |
| --- | --- | --- | --- | --- |
| D_0_ | 423,0620 | 481,2850 | 468,4350 | 457,5940^b^ |
| D_1_ | 471,2370 | 407,1750 | 453,7500 | 444,0540^d^ |
| D_2_ | 546,8550 | 352,4370 | 445,4790 | 448,2570^c^ |
| D_3_ | 720,2530 | 359,2840 | 470,2680 | 516,6017^a^ |
| D_4_ | 330,3750 | 300,4590 | 425,4120 | 352,0820^e^ |
| Total | 498,3564^a^ | 380,1280^c^ | 452,6688^b^ | 443,7177 |

*The differences between the averages shown with different letters in the columns and rows are significant at the rate of 0.01.
