# Supplementary material for: Seed priming with essential oils for sustainable wheat agriculture in semi-arid region
Source: PeerJ. 2023 Mar 27;11:e15126. doi: 10.7717/peerj.15126 (PMC10062347; doi:10.7717/peerj.15126)
Supplement: Supplemental Information 5 [file peerj-11-15126-s005.docx]

| Essential Oil Type | Rep. | PN (m^2^/plant) | PHH  (cm) | SL  (cm) | SGN | SGY | GY (kg/da) | TGW  (g) |
| --- | --- | --- | --- | --- | --- | --- | --- | --- |
| 1,00 | 1 | 446 | 85,28 | 7 | 21 | 7 | 256,6 | 42,3 |
| 1,00 | 2 | 445 | 81,21 | 9,5 | 21 | 8 | 256,5 | 43,2 |
| 1,00 | 3 | 447 | 86,42 | 10 | 19 | 6 | 256,4 | 41,5 |
| 1,00 | 4 | 445 | 82,71 | 10 | 12 | 5 | 256,3 | 42,9 |
| 1,00 | 5 | 448 | 88,34 | 9 | 18 | 9 | 256,7 | 44,5 |
| 1,00 | 6 | 446 | 80,46 | 8 | 23 | 10 | 256,8 | 45,6 |
| 1,00 | 7 | 444 | 87,31 | 9 | 17 | 8 | 256,9 | 43,4 |
| 1,00 | 8 | 443 | 84,76 | 10,5 | 19 | 7 | 256,2 | 41,6 |
| 1,00 | 9 | 448 | 85,94 | 10,5 | 14 | 9 | 256,1 | 44,2 |
| 1,00 | 10 | 444 | 86,34 | 9,5 | 15 | 6 | 256,7 | 43,8 |
| 2,00 | 1 | 432 | 81,33 | 9 | 14 | 8 | 234,4 | 40,2 |
| 2,00 | 2 | 431 | 79,12 | 9 | 25 | 9 | 234,3 | 40,3 |
| 2,00 | 3 | 430 | 81,91 | 10 | 20 | 8 | 234,5 | 41,5 |
| 2,00 | 4 | 433 | 82,13 | 10,5 | 24 | 9 | 234,4 | 39,5 |
| 2,00 | 5 | 434 | 83,46 | 9,5 | 21 | 9 | 234,6 | 38,4 |
| 2,00 | 6 | 435 | 80,46 | 8 | 31 | 8 | 234,7 | 42,1 |
| 2,00 | 7 | 430 | 79,37 | 9,5 | 18 | 9 | 234,2 | 40,5 |
| 2,00 | 8 | 431 | 79,16 | 9 | 21 | 10 | 234,0 | 41,0 |
| 2,00 | 9 | 431 | 81,08 | 8,5 | 13 | 8 | 234,1 | 37,9 |
| 2,00 | 10 | 430 | 79,91 | 8 | 20 | 8 | 234,4 | 40,0 |
| 3,00 | 1 | 428 | 91,81 | 10 | 18 | 6 | 224,8 | 40,1 |
| 3,00 | 2 | 429 | 93,29 | 10 | 18 | 5 | 224,9 | 38,5 |
| 3,00 | 3 | 430 | 92,41 | 9,5 | 16 | 8 | 224,7 | 39,1 |
| 3,00 | 4 | 427 | 90,13 | 9 | 17 | 7 | 224,6 | 40,8 |
| 3,00 | 5 | 428 | 89,46 | 10,5 | 12 | 6 | 224,5 | 39,9 |
| 3,00 | 6 | 431 | 92,46 | 9 | 10 | 9 | 224,2 | 38,1 |
| 3,00 | 7 | 429 | 90,89 | 10 | 18 | 5 | 224,1 | 37,6 |
| 3,00 | 8 | 428 | 93,49 | 9 | 22 | 6 | 224,8 | 39,4 |
| 3,00 | 9 | 431 | 94,15 | 9 | 20 | 5 | 224,8 | 41,2 |
| 3,00 | 10 | 430 | 90,56 | 9,5 | 18 | 8 | 224,8 | 41,1 |
| 4,00 | 1 | 430 | 98,75 | 10 | 26 | 6 | 219,2 | 37,6 |
| 4,00 | 2 | 431 | 101,34 | 8 | 17 | 5 | 219,3 | 34,6 |
| 4,00 | 3 | 432 | 99,64 | 9 | 17 | 8 | 219,6 | 35,5 |
| 4,00 | 4 | 434 | 98,12 | 8,5 | 19 | 7 | 219,5 | 36,4 |
| 4,00 | 5 | 428 | 97,46 | 8,5 | 22 | 6 | 219,7 | 35,9 |
| 4,00 | 6 | 429 | 100,76 | 9 | 17 | 5 | 219,4 | 38,0 |
| 4,00 | 7 | 427 | 97,64 | 9 | 16 | 6 | 219,2 | 33,4 |
| 4,00 | 8 | 430 | 102,84 | 10 | 18 | 6 | 219,3 | 35,0 |
| 4,00 | 9 | 431 | 99,46 | 10,5 | 18 | 7 | 219,0 | 36,6 |
| 4,00 | 10 | 429 | 98,53 | 12 | 19 | 8 | 219,2 | 37,9 |

PN: Number of plants per square meter, PHH: Plant height at harvest period, SL: Spike length, SGN: Number of grains per spike, SGY: Spike grain yield, GY: Grain yield per unit area, TGW: Thousand-grain weight

Type of E.oil: 1: Rosemary; 2: Sage, 3: Lavander
