# Supplementary material for: Seed priming with essential oils for sustainable wheat agriculture in semi-arid region
Source: PeerJ. 2023 Mar 27;11:e15126. doi: 10.7717/peerj.15126 (PMC10062347; doi:10.7717/peerj.15126)
Supplement: Supplemental Information 6 [file peerj-11-15126-s006.docx]

| E.O.  Dose | type of E.oil | Repetition | Germination (%) | coleoptil length (cm) | Shoot  Length  (cm) | Root length  (cm) | RWC  (%) | Chlorophyll  total | proline |
| --- | --- | --- | --- | --- | --- | --- | --- | --- | --- |
| 1,00 | 1 | 1 | 92 | 6 | 19 | 11 | 70,31 | 423,07 | 2,49 |
| 1,00 | 1 | 2 | 89 | 5 | 16 | 9,5 | 69,78 | 423,05 | 2,50 |
| 1,00 | 1 | 3 | 91 | 5,5 | 16,5 | 8 | 70,46 | 423,06 | 2,48 |
| 1,00 | 1 | 4 | 91 | 5 | 17 | 8,5 | 70,69 | 423,07 | 2,49 |
| 1,00 | 1 | 5 | 93 | 4,5 | 18 | 12 | 71,10 | 423,05 | 2,50 |
| 1,00 | 1 | 6 | 93 | 6 | 17 | 10 | 69,46 | 423,06 | 2,48 |
| 1,00 | 1 | 7 | 91 | 4,5 | 16 | 10,5 | 69,81 | 423,07 | 2,49 |
| 1,00 | 1 | 8 | 92 | 5 | 15 | 11 | 70,84 | 423,07 | 2,50 |
| 1,00 | 1 | 9 | 91 | 5 | 19,5 | 10,5 | 70,57 | 423,05 | 2,48 |
| 1,00 | 1 | 10 | 91 | 5 | 16,5 | 8,5 | 70,84 | 423,07 | 2,49 |
| 1,00 | 2 | 1 | 90 | 6,5 | 20 | 12 | 69,34 | 481,296 | 2,42 |
| 1,00 | 2 | 2 | 91 | 5,5 | 18 | 11 | 69,13 | 481,286 | 2,41 |
| 1,00 | 2 | 3 | 91 | 6 | 16 | 10 | 69,46 | 481,276 | 2,43 |
| 1,00 | 2 | 4 | 89 | 5,5 | 18 | 10,5 | 68,98 | 481,296 | 2,42 |
| 1,00 | 2 | 5 | 89 | 5,5 | 17 | 12,5 | 68,41 | 481,286 | 2,41 |
| 1,00 | 2 | 6 | 93 | 5 | 10 | 8,5 | 70,13 | 481,276 | 2,43 |
| 1,00 | 2 | 7 | 91 | 5,5 | 19 | 8,5 | 69,73 | 481,296 | 2,42 |
| 1,00 | 2 | 8 | 92 | 5 | 17 | 8 | 68,94 | 481,286 | 2,41 |
| 1,00 | 2 | 9 | 91 | 6,5 | 16 | 9 | 69,16 | 481,276 | 2,43 |
| 1,00 | 2 | 10 | 93 | 5,5 | 19,5 | 8 | 70,21 | 481,276 | 2,42 |
| 1,00 | 3 | 1 | 90 | 5 | 16 | 13 | 70,69 | 468,444 | 2,19 |
| 1,00 | 3 | 2 | 89 | 4 | 18 | 12 | 71,11 | 468,434 | 2,20 |
| 1,00 | 3 | 3 | 91 | 5 | 17 | 10,5 | 68,93 | 468,424 | 2,18 |
| 1,00 | 3 | 4 | 91 | 4,5 | 15,5 | 11 | 69,16 | 468,444 | 2,19 |
| 1,00 | 3 | 5 | 89 | 5 | 16,5 | 12 | 69,10 | 468,434 | 2,20 |
| 1,00 | 3 | 6 | 93 | 5,5 | 15,5 | 11 | 69,48 | 468,424 | 2,18 |
| 1,00 | 3 | 7 | 89 | 6 | 18 | 10 | 70,31 | 468,444 | 2,19 |
| 1,00 | 3 | 8 | 92 | 4,5 | 18,5 | 10,5 | 69,74 | 468,434 | 2,20 |
| 1,00 | 3 | 9 | 91 | 4,5 | 16,5 | 10 | 69,88 | 468,424 | 2,18 |
| 1,00 | 3 | 10 | 91 | 5 | 17,5 | 9,5 | 70,82 | 468,444 | 2,19 |
| 2,00 | 1 | 1 | 89 | 7 | 20 | 14 | 71,33 | 471,238 | 2,46 |
| 2,00 | 1 | 2 | 92 | 5 | 17,5 | 11 | 71,32 | 471,248 | 2,47 |
| 2,00 | 1 | 3 | 87 | 8 | 18 | 13,5 | 71,31 | 471,228 | 2,45 |
| 2,00 | 1 | 4 | 88 | 8,5 | 21 | 12 | 70,46 | 471,238 | 2,46 |
| 2,00 | 1 | 5 | 91 | 5,5 | 19 | 15 | 71,64 | 471,248 | 2,47 |
| 2,00 | 1 | 6 | 88 | 8 | 16,5 | 9,5 | 70,86 | 471,228 | 2,45 |
| 2,00 | 1 | 7 | 87 | 5 | 18 | 11 | 70,76 | 471,238 | 2,46 |
| 2,00 | 1 | 8 | 89 | 6,5 | 17,5 | 13 | 70,55 | 471,248 | 2,47 |
| 2,00 | 1 | 9 | 92 | 4,5 | 19 | 14 | 71,08 | 471,228 | 2,45 |
| 2,00 | 1 | 10 | 91 | 6,5 | 20,5 | 12,5 | 71,17 | 471,228 | 2,46 |
| 2,00 | 2 | 1 | 94 | 6 | 24 | 15 | 71,16 | 407,184 | 2,51 |
| 2,00 | 2 | 2 | 96 | 7 | 21 | 12 | 69,84 | 407,174 | 2,52 |
| 2,00 | 2 | 3 | 92 | 7 | 20,5 | 10,5 | 69,76 | 407,164 | 2,50 |
| 2,00 | 2 | 4 | 95 | 8 | 19,5 | 16,5 | 71,46 | 407,184 | 2,51 |
| 2,00 | 2 | 5 | 93 | 7,5 | 21 | 14 | 70,16 | 407,174 | 2,52 |
| 2,00 | 2 | 6 | 92 | 6,5 | 19,5 | 15,5 | 70,34 | 407,164 | 2,50 |
| 2,00 | 2 | 7 | 94 | 7 | 20,5 | 14 | 70,12 | 407,184 | 2,51 |
| 2,00 | 2 | 8 | 96 | 8,5 | 20 | 13,5 | 69,46 | 407,174 | 2,52 |
| 2,00 | 2 | 9 | 93 | 8 | 19 | 14 | 69,81 | 407,164 | 2,50 |
| 2,00 | 2 | 10 | 95 | 6 | 19,5 | 15 | 70,21 | 407,184 | 2,51 |
| 2,00 | 3 | 1 | 92 | 5 | 16 | 13 | 70,12 | 453,84 | 2,01 |
| 2,00 | 3 | 2 | 91 | 6,5 | 13 | 12 | 70,34 | 453,74 | 2,00 |
| 2,00 | 3 | 3 | 87 | 6 | 13,5 | 10,5 | 69,84 | 453,64 | 2,02 |
| 2,00 | 3 | 4 | 89 | 5 | 15 | 14 | 69,99 | 453,84 | 2,01 |
| 2,00 | 3 | 5 | 92 | 5,5 | 14,5 | 13 | 70,42 | 453,74 | 2,00 |
| 2,00 | 3 | 6 | 88 | 6 | 16 | 11 | 68,94 | 453,64 | 2,02 |
| 2,00 | 3 | 7 | 87 | 6,5 | 15 | 10,5 | 69,34 | 453,84 | 2,01 |
| 2,00 | 3 | 8 | 91 | 5 | 15,5 | 10 | 69,98 | 453,74 | 2,00 |
| 2,00 | 3 | 9 | 88 | 5,5 | 14 | 9,5 | 70,98 | 453,64 | 2,02 |
| 2,00 | 3 | 10 | 89 | 5 | 13 | 10 | 70,84 | 453,84 | 2,01 |
| 3,00 | 1 | 1 | 94 | 7 | 25 | 15,5 | 75,64 | 546,854 | 2,05 |
| 3,00 | 1 | 2 | 93 | 8,5 | 21 | 12,5 | 75,10 | 546,844 | 2,04 |
| 3,00 | 1 | 3 | 96 | 7,5 | 19,5 | 13 | 75,79 | 546,864 | 2,06 |
| 3,00 | 1 | 4 | 92 | 7 | 23 | 14,5 | 74,36 | 546,854 | 2,05 |
| 3,00 | 1 | 5 | 93 | 6,5 | 22 | 13,5 | 76,21 | 546,844 | 2,04 |
| 3,00 | 1 | 6 | 92 | 7 | 24 | 13,5 | 74,98 | 546,864 | 2,06 |
| 3,00 | 1 | 7 | 94 | 7,5 | 21 | 14 | 74,56 | 546,854 | 2,05 |
| 3,00 | 1 | 8 | 92 | 6,5 | 21,5 | 12,5 | 76,04 | 546,844 | 2,04 |
| 3,00 | 1 | 9 | 93 | 7 | 22,5 | 13 | 75,34 | 546,864 | 2,06 |
| 3,00 | 1 | 10 | 94 | 6,5 | 24,5 | 15 | 75,19 | 546,864 | 2,05 |
| 3,00 | 2 | 1 | 93 | 7 | 19,5 | 15 | 72,21 | 352,428 | 2,24 |
| 3,00 | 2 | 2 | 96 | 6,5 | 20,5 | 16 | 72,96 | 352,438 | 2,25 |
| 3,00 | 2 | 3 | 94 | 6 | 21 | 13,5 | 71,84 | 352,448 | 2,23 |
| 3,00 | 2 | 4 | 95 | 7 | 18,5 | 16,5 | 71,64 | 352,428 | 2,24 |
| 3,00 | 2 | 5 | 92 | 6 | 18,5 | 14 | 71,36 | 352,438 | 2,25 |
| 3,00 | 2 | 6 | 94 | 7 | 19 | 15,5 | 72,09 | 352,448 | 2,23 |
| 3,00 | 2 | 7 | 95 | 6,5 | 18,5 | 16 | 72,51 | 352,428 | 2,24 |
| 3,00 | 2 | 8 | 93 | 6,5 | 19 | 15,5 | 71,34 | 352,438 | 2,25 |
| 3,00 | 2 | 9 | 96 | 7 | 17,5 | 16 | 71,98 | 352,448 | 2,23 |
| 3,00 | 2 | 10 | 92 | 6,5 | 20 | 14,5 | 71,24 | 352,428 | 2,24 |
| 3,00 | 3 | 1 | 95 | 6 | 15 | 13 | 71,12 | 445,488 | 2,24 |
| 3,00 | 3 | 2 | 93 | 5 | 13 | 14 | 72,13 | 445,478 | 2,23 |
| 3,00 | 3 | 3 | 90 | 5,5 | 12,5 | 11 | 71,34 | 445,468 | 2,25 |
| 3,00 | 3 | 4 | 92 | 6 | 14 | 10,5 | 71,09 | 445,488 | 2,24 |
| 3,00 | 3 | 5 | 93 | 4,5 | 16 | 11,5 | 71,46 | 445,478 | 2,23 |
| 3,00 | 3 | 6 | 92 | 5 | 15 | 12 | 71,84 | 445,468 | 2,25 |
| 3,00 | 3 | 7 | 92 | 5,5 | 14,5 | 13 | 70,64 | 445,488 | 2,24 |
| 3,00 | 3 | 8 | 94 | 6 | 13,5 | 13,5 | 71,48 | 445,478 | 2,23 |
| 3,00 | 3 | 9 | 90 | 5 | 14 | 14 | 70,89 | 445,468 | 2,25 |
| 3,00 | 3 | 10 | 93 | 5,5 | 12 | 11 | 72,84 | 445,488 | 2,24 |
| 4,00 | 1 | 1 | 84 | 3 | 21 | 16 | 72,16 | 720,254 | 2,09 |
| 4,00 | 1 | 2 | 81 | 2 | 22 | 13,5 | 71,66 | 720,244 | 2,10 |
| 4,00 | 1 | 3 | 82 | 2,5 | 17,5 | 15,5 | 71,46 | 720,264 | 2,08 |
| 4,00 | 1 | 4 | 84 | 3 | 18 | 14,5 | 71,31 | 720,254 | 2,09 |
| 4,00 | 1 | 5 | 81 | 2,5 | 19,5 | 13 | 72,01 | 720,244 | 2,10 |
| 4,00 | 1 | 6 | 80 | 1,5 | 18 | 13 | 71,82 | 720,264 | 2,08 |
| 4,00 | 1 | 7 | 81 | 2,5 | 21 | 16,5 | 71,13 | 720,254 | 2,09 |
| 4,00 | 1 | 8 | 80 | 2 | 20,5 | 12,5 | 70,64 | 720,244 | 2,10 |
| 4,00 | 1 | 9 | 81 | 2 | 19 | 15 | 70,84 | 720,264 | 2,08 |
| 4,00 | 1 | 10 | 82 | 2 | 18,5 | 13,5 | 70,69 | 720,244 | 2,09 |
| 4,00 | 2 | 1 | 87 | 5 | 13,5 | 15 | 67,16 | 359,292 | 2,39 |
| 4,00 | 2 | 2 | 85 | 4,5 | 16,5 | 13 | 67,94 | 359,282 | 2,38 |
| 4,00 | 2 | 3 | 86 | 4,5 | 17 | 14 | 68,21 | 359,272 | 2,40 |
| 4,00 | 2 | 4 | 85 | 4,5 | 14,5 | 15 | 67,34 | 359,292 | 2,39 |
| 4,00 | 2 | 5 | 87 | 5 | 14,5 | 16 | 67,49 | 359,282 | 2,38 |
| 4,00 | 2 | 6 | 84 | 6 | 13,5 | 13 | 68,10 | 359,292 | 2,40 |
| 4,00 | 2 | 7 | 86 | 4,5 | 14 | 13,5 | 67,09 | 359,282 | 2,39 |
| 4,00 | 2 | 8 | 85 | 5,5 | 13 | 12,5 | 68,23 | 359,272 | 2,38 |
| 4,00 | 2 | 9 | 85 | 5 | 13,5 | 14 | 67,38 | 359,292 | 2,40 |
| 4,00 | 2 | 10 | 84 | 5,5 | 15,5 | 15 | 67,84 | 359,282 | 2,39 |
| 4,00 | 3 | 1 | 76 | 5 | 15 | 10 | 70,69 | 470,268 | 2,20 |
| 4,00 | 3 | 2 | 81 | 4,5 | 12,5 | 9,5 | 69,45 | 470,278 | 2,19 |
| 4,00 | 3 | 3 | 78 | 4,5 | 13,5 | 8 | 68,13 | 470,258 | 2,21 |
| 4,00 | 3 | 4 | 77 | 4,5 | 14 | 11 | 68,05 | 470,268 | 2,20 |
| 4,00 | 3 | 5 | 78 | 3 | 15 | 10 | 69,31 | 470,278 | 2,19 |
| 4,00 | 3 | 6 | 80 | 6 | 16 | 9,5 | 69,46 | 470,258 | 2,21 |
| 4,00 | 3 | 7 | 76 | 4,5 | 13 | 8 | 70,43 | 470,268 | 2,20 |
| 4,00 | 3 | 8 | 77 | 5,5 | 12,5 | 11 | 68,16 | 470,278 | 2,19 |
| 4,00 | 3 | 9 | 81 | 5 | 12 | 10 | 70,13 | 470,258 | 2,21 |
| 4,00 | 3 | 10 | 80 | 3,5 | 13 | 8,5 | 69,45 | 470,268 | 2,20 |
| 5,00 | 1 | 1 | 43 | 3 | 13 | 12,5 | 70,91 | 330,374 | 2,52 |
| 5,00 | 1 | 2 | 42 | 2 | 10,5 | 9,5 | 70,68 | 330,364 | 2,51 |
| 5,00 | 1 | 3 | 45 | 2,5 | 9,5 | 9 | 71,16 | 330,384 | 2,53 |
| 5,00 | 1 | 4 | 41 | 3 | 12 | 10,5 | 71,31 | 330,374 | 2,52 |
| 5,00 | 1 | 5 | 42 | 2,5 | 13 | 13,5 | 70,84 | 330,364 | 2,51 |
| 5,00 | 1 | 6 | 39 | 1,5 | 10,5 | 8,5 | 71,23 | 330,384 | 2,53 |
| 5,00 | 1 | 7 | 42 | 2,5 | 11 | 10 | 70,64 | 330,374 | 2,52 |
| 5,00 | 1 | 8 | 41 | 2 | 9,5 | 9,5 | 71,11 | 330,364 | 2,51 |
| 5,00 | 1 | 9 | 42 | 2 | 10 | 9 | 70,46 | 330,384 | 2,53 |
| 5,00 | 1 | 10 | 40 | 2 | 11 | 10 | 70,31 | 330,384 | 2,52 |
| 5,00 | 2 | 1 | 40 | 3 | 10,5 | 14 | 64,64 | 300,468 | 2,10 |
| 5,00 | 2 | 2 | 42 | 2 | 8 | 12 | 63,40 | 300,458 | 2,11 |
| 5,00 | 2 | 3 | 40 | 3 | 7,5 | 13,5 | 63,76 | 300,448 | 2,09 |
| 5,00 | 2 | 4 | 41 | 2,5 | 9 | 10 | 63,89 | 300,468 | 2,10 |
| 5,00 | 2 | 5 | 42 | 1,5 | 9 | 9,5 | 64,34 | 300,458 | 2,11 |
| 5,00 | 2 | 6 | 39 | 2 | 9,5 | 10,5 | 64,49 | 300,448 | 2,09 |
| 5,00 | 2 | 7 | 42 | 1,5 | 11 | 8,5 | 63,78 | 300,468 | 2,10 |
| 5,00 | 2 | 8 | 41 | 2 | 9 | 10,5 | 63,47 | 300,458 | 2,11 |
| 5,00 | 2 | 9 | 42 | 1,5 | 12 | 11 | 64,10 | 300,448 | 2,09 |
| 5,00 | 2 | 10 | 40 | 2 | 8,5 | 11,5 | 64,08 | 300,468 | 2,10 |
| 5,00 | 3 | 1 | 40 | 1,5 | 13 | 9 | 69,12 | 425,412 | 2,08 |
| 5,00 | 3 | 2 | 39 | 2 | 12 | 8,5 | 68,42 | 425,422 | 2,09 |
| 5,00 | 3 | 3 | 40 | 3 | 11,5 | 11 | 68,79 | 425,402 | 2,07 |
| 5,00 | 3 | 4 | 41 | 2,5 | 13 | 10,5 | 67,16 | 425,412 | 2,08 |
| 5,00 | 3 | 5 | 42 | 2,5 | 13,5 | 9 | 67,46 | 425,422 | 2,09 |
| 5,00 | 3 | 6 | 42 | 2 | 12 | 8,5 | 67,81 | 425,402 | 2,07 |
| 5,00 | 3 | 7 | 42 | 1,5 | 10 | 10,5 | 67,14 | 425,412 | 2,08 |
| 5,00 | 3 | 8 | 41 | 2 | 10,5 | 9 | 68,31 | 425,422 | 2,09 |
| 5,00 | 3 | 9 | 40 | 1,5 | 11 | 8,5 | 67,84 | 425,402 | 2,07 |
| 5,00 | 3 | 10 | 42 | 2 | 10,5 | 9 | 68,09 | 425,412 | 2,08 |

Essential oil doses: 1,00: D_0_ (CONTROL); 2,00: D_1_ (%0,01); 3,00: D_2_(%0,05); 4,00: D_3_ (%0,10); 5,00: D_4_ (%0,25)

Type of E.oil: 1: Rosemary; 2: Sage, 3: Lavander
